# Morphological and Physiological Changes in Mature *In Vitro* Neuronal Networks towards Exposure to Short-, Middle- or Long-Term Simulated Microgravity

**DOI:** 10.1371/journal.pone.0073857

**Published:** 2013-09-16

**Authors:** Giuseppe Pani, Nada Samari, Roel Quintens, Louis de Saint-Georges, MariAntonia Meloni, Sarah Baatout, Patrick Van Oostveldt, Mohammed Abderrafi Benotmane

**Affiliations:** 1 Radiobiology Unit, Molecular and Cellular Biology expert group, Belgian Nuclear Research Centre, SCK•CEN, Mol, Belgium; 2 Laboratory of Biochemistry and Molecular Cytology, Department of Molecular Biotechnology, Ghent University, Ghent, Belgium; 3 Department of Physiological, Biochemical and Cellular Science, University of Sassari, Sassari, Italy; Charité University Medicine Berlin, Germany

## Abstract

One of the objectives of the current international space programmes is to investigate the possible effects of the space environment on the crew health. The aim of this work was to assess the particular effects of simulated microgravity on mature primary neuronal networks and specially their plasticity and connectivity. For this purpose, primary mouse neurons were first grown for 10 days as a dense network before being placed in the Random Positioning Machine (RPM), simulating microgravity. These cultures were then used to investigate the impact of short- (1 h), middle- (24 h) and long-term (10 days) exposure to microgravity at the level of neurite network density, cell morphology and motility as well as cytoskeleton properties in established two-dimensional mature neuronal networks. Image processing analysis of dense neuronal networks exposed to simulated microgravity and their subsequent recovery under ground conditions revealed different neuronal responses depending on the duration period of exposure. After short- and middle-term exposures to simulated microgravity, changes in neurite network, neuron morphology and viability were observed with significant alterations followed by fast recovery processes. Long exposure to simulated microgravity revealed a high adaptation of single neurons to the new gravity conditions as well as a partial adaptation of neuronal networks. This latter was concomitant to an increase of apoptosis. However, neurons and neuronal networks exposed for long-term to simulated microgravity required longer recovery time to re-adapt to the ground gravity. In conclusion, a clear modulation in neuronal plasticity was evidenced through morphological and physiological changes in primary neuronal cultures during and after simulated microgravity exposure. These changes were dependent on the duration of exposure to microgravity.

## Introduction

In an orbital spaceflight, astronauts are exposed to the orbital gravity (10^−2^–10^−6^×*g*), also called microgravity, which is a continuous free-fall condition, resulting from the Earth’s gravitational pull and the centrifugal forces from the spacecraft’s propulsion. Microgravity is one of the main stressful components of the space environment since it is well known that it induces physiological changes in astronauts such as skeletal muscle atrophy [1], bone loss [2], immune system impairment [3], [4] and shifts of body fluids from the lower extremities to the upper body [5]. Moreover, cognitive deficits, sensory-motor alterations, changes in sleep-wake regulation as well as vegetative disorders can also occur during long-term space flight, affecting human performance [6]. It is also known that organisms exposed to microgravity undergo physiological, cellular as well as metabolic changes. For instance, cellular motility, morphology, cytoskeleton [7], proliferation [8], apoptosis [9], [10] as well as other physiological systems are known to be altered following exposure to modified gravitational fields.

When astronauts and/or animals are exposed to microgravity, a particular number of neurological disorders, such as space adaptation syndrome (SAS), space motion sickness (SMS), postural illusion, visual disturbances, nausea and headaches, neuromuscular fatigue and weakness as well as postural imbalance and ataxia may appear and persist until return to Earth [11]. These pathological changes affect both motor and sensory functions, and the effects can be long lasting. Furthermore, it has been suggested that these changes could be the signs of an active process of neuroplasticity [12]. However, the nature of the functional and structural mechanisms involved in these changes is currently not well understood.

Basically, the term “neuroplasticity” is related to the neuronal capability to modify some functional processes in response to the alterations in incoming information [13]. It is an intrinsic property of the nervous system maintained throughout life that allows physiological modifications of neuron functions and structures in response to environmental changes via the strengthening, weakening, pruning or the addition of synaptic connections and/or the promotion of neurogenesis [14]. Increases in neuroplastic activity seem also to be linked to pain hypersensitivity and to headaches [14], [15]. Furthermore, it has been previously shown that environmental changes can alter cognition and behavior by modifying connections between existing neurons in the hippocampus, cortex and other parts of the brain [16].

Several experiments on cell morphology and motility have been performed in real and simulated microgravity. In these experiments loss of cell adhesion, reduced cell surface, decrease in the number of filopodia and reduced motility were reported [7], [17]. These functions are mainly regulated by cytoskeletal activities and the observed alterations concern in particular the distribution and organization of microtubules, microfilaments and the structure of adhesion proteins [7], [17], [18]. Alterations of microtubule and microfilament organization under simulated microgravity have already been evidenced in neuron outgrowth cones [19]. It was also reported that glial cells showed morphological alterations already after 30 minutes of simulated microgravity, and after 20–32 h, presented an elevated cell death rate [20]. Moreover, neurons, exposed to simulated microgravity before plating, showed cell clustering and abnormal shapes after 24 h of culture in ground conditions [21]. Experiments on neuronal connections in simulated microgravity also suggested that synapse formation is sensitive to the gravitational vector [22], [23].

Apoptosis, or programmed cell death, occurs in all multicellular organisms and the initiation is induced by various stimuli such as changes in cellular homeostasis, binding of particular ligands to cell surface receptors, radiation and environmental stress factors [24]. In the brain, apoptosis is known to be partly induced by cytoskeleton disruption in hippocampal cells [25]. Apoptosis induced by microgravity, both *in vivo* and *in vitro*, was also described in several experiments related to the central nervous system (CNS) [10], [26].

As previously described, microgravity can directly influence several parts of the CNS inducing a re-organization of neuron connections in order to codify the new inputs coming from the sensory systems. However, it is still unknown whether microgravity also exerts an effect on the CNS areas that are not directly involved with either the sensing or the response to gravity. In these CNS areas microgravity could induce alterations at the cellular level affecting thereafter events involved in neuronal plasticity and connectivity [6]. However, although some experiments reported morphological alterations in neurons cultured in altered gravity force [19], [21], [22], [27], until now only few *in vivo* and *in vitro* studies on mature nervous system models have been conducted to investigate the effects of real or simulated microgravity on adult neural plasticity processes [22], [27]. Results from the few *in vivo* studies on the effects of real or simulated space conditions on the CNS plasticity suggest that exposure to gravity alterations, both during microgravity as well as after return to Earth, induce changes in the mature nervous system [28]. During the Cosmos 1514 flight, rat pups were exposed to space conditions *in utero* and brains were thereafter morphologically and histochemically examined [29]. Ultrastructural studies revealed some delay in neuroblastic differentiation as well as in cytoskeletal changes in unmyelinated fibers and in outgrowth cones of axons and dendrites in the hypothalamic supraoptic nuclei [29]. Furthermore, experiments performed on rats during the Space Flight Science 1 and 2 reported changes in ribbon synaptic plasticity. In particular, it was demonstrated that gravity sensor hair cells have an extraordinary ability to change number, type and distribution of synapses [30]. Recently, a payload for rodents, named Mice Drawer System (MDS) was built to house mice aboard the International Space Station (ISS) for investigating the long-term adaptation to space conditions [31]. It was reported that the expression of neuron growth factor (NGF) and brain-derived neurotrophic factor (BDNF) was reduced in brain regions such as the cortex and the hippocampus of spaceflown animals as compared to ground control ones [32]. The same study revealed that genes involved in long-term potentiation, axon guidance, neuronal growth, cone collapse, cell migration, dendrite branching and dendritic-spine morphology were up-regulated in the whole brain of mice exposed for 91 days to the ISS environment [32].

In this study we investigated the effects of simulated microgravity using the Random Positioning Machine (RPM) on *in vitro* dense mature neuronal networks obtained from primary mouse neurons with a particular emphasis on neuronal network morphology and cell death during short-, middle and long-term exposure to simulated microgravity.

## Materials and Methods

### Primary Cell Cultures and Adult Neuronal Network Model

In this study, primary neuron cultures were initiated from brain cortex of 17 day-old mouse fetuses. All animal experiments were carried out in strict accordance with the recommendations from the Guide for the Care and Use of Laboratory Animals of the National Institutes of Health (USA). The protocol was approved by the SCK•CEN (Belgian Nuclear Research Centre, Mol, Belgium) and VITO (Flemish Institute for Technological Research, Geel, Belgium) Ethical Committee for Laboratory Animal Experimentation (Permit Number: 08-001). Three pregnant BALB/c mice, one per replicate, were sacrificed by cervical dislocation at day 17 post-conception. Subsequently, brains from mouse fetuses were dissected and cortices were extracted. Brain cortices of *foeti* from the same pregnant female were pooled and considered as one replicate. Treatment with 0.1% trypsin (cat n° 15400, Gibco, Belgium) and 10 µg/ml DNAse I (cat n° 18068015, Gibco, Belgium) in phosphate buffered saline solution allowed to isolate single neuronal cells which were then collected after centrifugation. Finally, neurons from the three replicate pools were seeded each in 18 4-well plates (54 4-well plates in total) (cat n° 76740, Thermo Scientific, Belgium) at a density of 50,000 cells per cm^2^. Neurons were plated in poly-D-lysine pre-coated wells (cat n° P0899, Sigma-Aldrich, Belgium) with MEM medium (cat n° 31095, Gibco, Belgium) supplemented with 10% fetal serum (cat n° 10437, Gibco) and penicillin-streptomycin (0.1%) (cat n° 15140, Gibco, Belgium) and incubated for 1 h at 37°C and 5% CO_2_ to allow adherence of neuron cells to the coated support. The medium was then exchanged for Neurobasal Medium (cat n° 10888-022, Gibco, Belgium) supplemented with 2% B27 supplement (cat n° 17504-044, Gibco, Belgium), HEPES 20 mM (cat n° 15630, Gibco, Belgium) and 0.2% penicillin-streptomycin. This selective medium stimulated the growth of neuronal cells present in the culture and inhibited other brain cell types.

In order to obtain dense and mature neuronal networks as *in vitro* model, neurons were cultured for 10 days at 37°C, 95% of humidity and 5% CO_2_. At days 5 and 7 of culture, 2/3 of each culture medium was replaced with fresh medium.

### *In vitro* Experimental Layout

To study the morphological effects of simulated microgravity on dense neuronal networks as well as on well-connected neurons, at day 9 of neuron culture, plates were prepared to be exposed to the desktop RPM (Dutch Space) for 1 hour (short-time exposure), 24 hours (middle-term exposure) and 10 days (long-term exposure). The complete experiment required fifty-four 4 well-plates, eighteen plates per replicate (9 GC and 9 RPM), which were fully filled with complete neurobasal medium and sealed with sterile parafilm. Bubbles were removed with a syringe. At day 10, 3×9 plates were exposed to the RPM at 60°/s (0.03–0.008×g) [33] for 1 h, 24 h or 10 days. For every time point, the three remaining plates were positioned on the static RPM bar as ground controls (GC). After 1 h, 24 h and 10 days of exposure, one plate per condition was fixed with 4% paraformaldehyde, whereas the remaining two were kept for 24 or 72 h at normal ground conditions to further investigate the neuronal recovery after simulated microgravity. The experimental layout is described in Fig. S1.

### Immunostaining of Neuronal Network

Neuronal networks were stained for the neuronal marker β-tubulin 3 (β-tub 3) using indirect immunofluorescence, whereas nuclei were revealed by direct fluorescence. After washing with phosphate buffered saline (PBS), cells were permeabilized with PBS-Triton X-100 (Sigma, Belgium) 0.1% for 3 min and blocked for 30 min with PBS-BSA 3%. Fluorescent staining was performed by exposing the samples to mouse monoclonal anti-β-tubulin 3 (cat n° T5076-200UL, Sigma-Aldrich, Belgium), diluted 1∶200 in PBS (Sigma, Belgium), at 4°C overnight. After washing in PBS, a second layer of fluorescein isothiocyanate (FITC)-conjugated anti-Mouse IgG (cat n°F2012, Sigma-Aldrich) antibody, diluted 1∶200 in PBS, was applied for 90 min, at 37°C in the dark. Nuclei were then stained with Hoechst (cat n° B2883, Sigma-Aldrich), 1∶400 in PBS, for 10 min. Wells were then rinsed three times with PBS and twice with H_2_O.

### Image Acquisition and Neuronal Network Analysis

Images were acquired with a Nikon Eclipse Ti (automated inverted wide-field epifluorescence microscope) equipped with a 40×magnification (S PLAN FLUR, ELWD 40x/0. 6) dry objective and a Nikon DS-Qi1Mc camera controlled by a NIS-Elements software.

Per each condition, three mosaic regions of 3 by 3 images (27 bi-dimensional images, in total) with five focus positions were randomly acquired and compressed in a 2D in focus image by Extended Depth of Focus (EDF) NIS-Elements module. Data obtained from the 3 mosaics were summed and considered as a single large image.

The neuronal network image processing analyses were performed by a home-made tool for ImageJ (Rasband, W.S., N.I.H, USA, http://rsb.info.nih.gov/ij/) [34] by merging and implementing known functions available in the freely available plugin [35]–[38]. The high performance of this new tool came from an appropriate soma segmentation originating from nuclei and from an elegant multi-tier segmentation after image enhancing and edge detection, segmenting even the thinner neurites. Thereafter, morphological processes could be easily applied for the determination of the total neuron area, the total neurite area and length, the neurite attachment points, the soma counting and soma characteristics. Data related to area and length of neurites per image were determined to specifically investigate the effects of simulated microgravity on the neurite network density *in toto*. Finally, morphological values per single neuron were obtained dividing image parameters by number of neurons per image in order to obtain an average per cell.

In order to better understand the distribution of β-tub 3, the mean intensity of fluorescence was determined in somas and neurites. Calculating the ratios between mean intensities of somas and neurites allowed us to determine the distribution of tubulin into neurons.

Each experiment was performed in triplicates and all statistical analyses were performed using GraphPad Prism for Windows (GraphPad Software, San Diego California USA, www.graphpad.com) Normal distribution of the obtained cytometric parameters (area of neurons, area and length of neurites estimated per image and per neurons, as well as soma size and shape and distribution of β-tub 3) was analyzed using Shapiro-Wilk’s test. For all these cytometric parameters the assumption of normality was not rejected. A significant difference from the control conditions was therefore determined using the parametric paired *t*-test or one-way ANOVA and a *p*-value <0.05 was considered significant.

### Apoptosis

Apoptosis was estimated by Annexin V (Ann V) assay on adherent neurons using the Ann V-FITC apoptosis detection kit II (cat n° BMS500FI/300CE, eBioscience, Belgium) combined with an additional fluorescence staining of nuclei by Hoechst dye. Ann V-FITC was used to quantitatively estimate the percentage of dead cells in the neuron cultures. The AnnV-FITC^−/^PI^−/^Hoechst^+^ population was considered as normal healthy cells, while Ann V-FITC^+^/PI^−/^Hoechst^+^ (Ann V^+^-PI^−^) and Ann V-FITC^+^/PI^+^/Hoechst^+^ (AnnV^+^-PI^+^) cells were taken as an estimation of early apoptosis and late apoptosis or necrosis, respectively. Image processing analysis is described in Fig. S2 and Fig. S3. Primary neuron cultures are not 100% pure cultures as a small number of non-neuron cells (negative to β-tub 3) with small nuclei and condensed chromatin were observed after nuclei and neuron marker staining. This type of cells were positive for PI and negative for Ann V staining and were therefore not taken into account in the viability estimation since they could induce a relative error in the counting of late apoptotic/necrotic neuron cells.

For statistical analysis, 36 images were randomly acquired with 40×objective and an average of 530±130 cells per condition were taken into account. Ann V^–^PI^−^ cells as well as the specific Ann V^+^-PI^−^ and Ann V^+^-PI^+^ cells were counted and the percentages of Ann V^+^-PI^−^ and Ann V^+^-PI^+^ neurons on total cell number were then calculated. To estimate the relative level of total Ann V^+^ (Ann V^+^-PI^−^+Ann V^+^-PI^+^) neurons exposed to simulated microgravity, the previous obtained percentages were divided by the percentage obtained in respective control cultures. Based on nucleus counterstaining of adherent neurons, the density of cells per cm^2^ was estimated in neuron cultures exposed for short-, middle- or long-term to RPM. This allowed to compare neuron cultures exposed to the RPM vs. their respective controls. Additionally, comparison between 1 h GC and 10 days RPM was performed to observe the loss of cells over the whole experiment. For comparison between conditions, parametric paired *t*-test using GraphPad Prism for Windows was performed after confirming the normal distribution of outcome parameters with Shapiro-Wilk’s test. Finally, *p*-value <0.05 was considered significant.

## Results

### Behavior of Neurons in Culture

**Figure 1:**
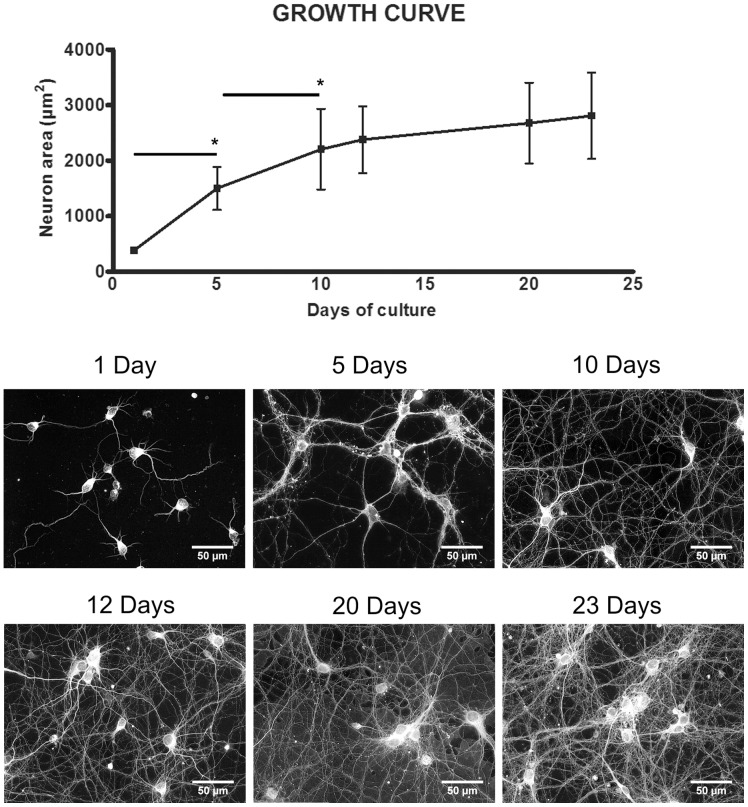
Neuronal network growth under ground conditions. Growth curve of the neuronal area (upper panel) within a period of 23 days with representative images at selective time points (lower panel). Values are expressed as the average of single neuron areas and bars represent standard deviation. Paired *t*-test was performed to determine differences between two close experimental points and obtained results showed significant differences between 1 day vs. 5 days (*p* = 0.046) and 5 days vs. 10 days (*p = *0.041) but not between the following time points.

Monitoring the growth of the neuronal network on mouse primary cortical neurons cultured under ground conditions up to 23 days allowed to determine at which stage a sufficient connectivity between neurons occurred indicating a good maturation of the neuronal network as previously demonstrated in other studies [39], [40]. As shown in figure 1 (Fig. 1) we observed that during the first ten days of culture the neuronal network grew fast; thereafter the growth rate slowed down. Therefore, we decided to use 10-day old cultures for subsequent experiments.

### Behavior of the Neuronal Networks under Simulated Microgravity

To investigate the effects of simulated microgravity on dense neuronal networks, 10-day cultures were exposed for short-, middle- and long-terms to the RPM. Immunostaining of β-tub as a specific marker of neurons was used to analyze the changes in the neuronal network.

#### Changes of neuron area and neurite area and length under simulated microgravity

**Figure 2:**
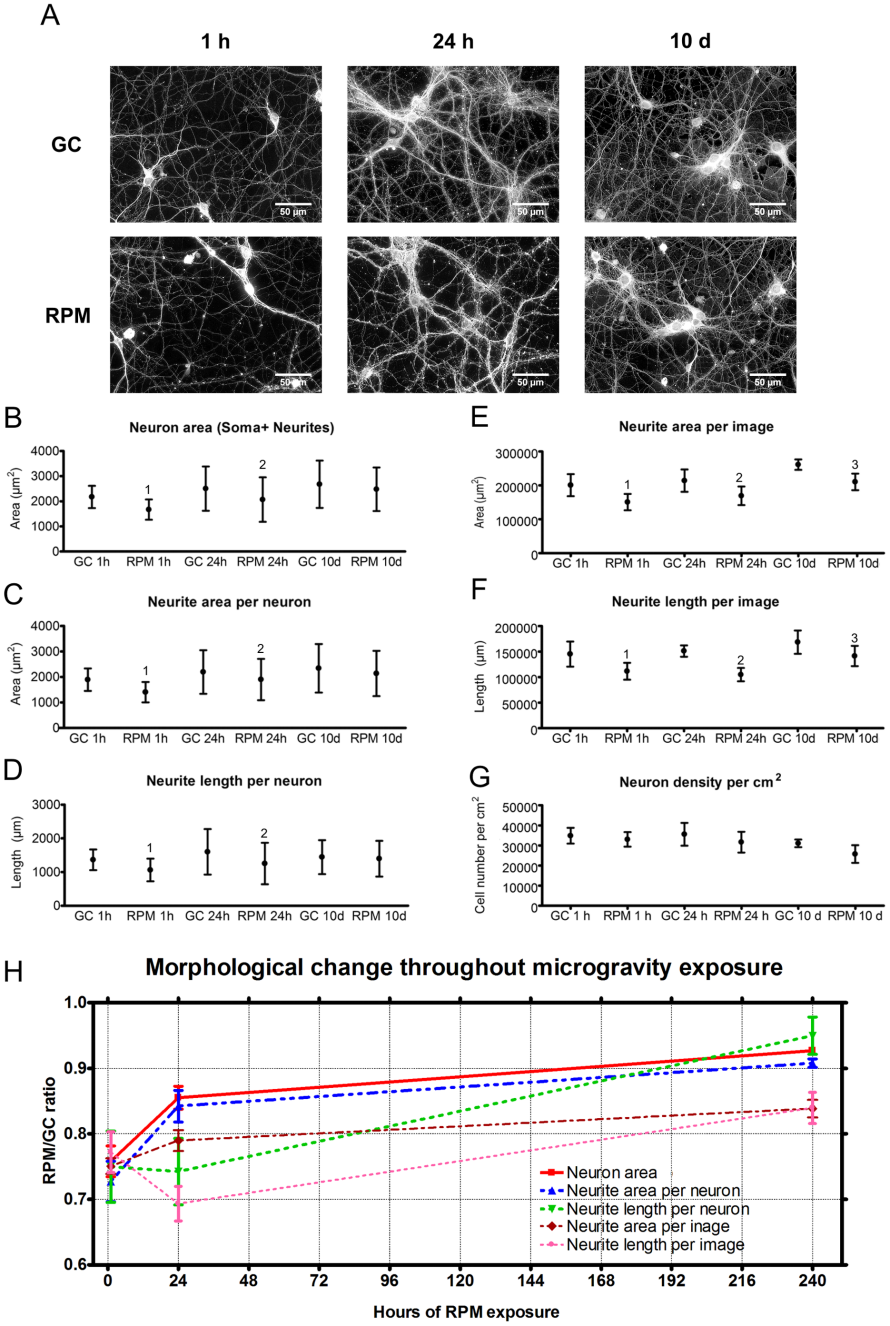
Effects of simulated microgravity on single neurons as well as on neuronal networks. (A) First line: neuronal networks cultured in ground control conditions (GC 1 h - 24 h - 10 days); second line: neuronal networks exposed to simulated microgravity (RPM 1 h - 24 h - 10 days). (B) Neuron area (soma+neurites) in neuronal network cultures exposed to RPM for 1 h, 24 h or 10 days and the respective controls. (C) Neurite area per neuron in neuronal network cultures exposed to RPM for 1 h, 24 h or 10 days and the respective controls. (D) Neurite length per neuron in neuronal network cultures exposed to RPM for 1 h, 24 h or 10 days and the respective controls. (E) Neurite area per image in cultures exposed to RPM for 1 h, 24 h or 10 days and the respective controls. (F) Neurite length per image in cultures exposed to RPM for 1 h, 24 h or 10 days and the respective controls. (G) Neuronal density per cm^2^; 10 day RPM vs. 1 h GC : *p = *0.055. (H) RPM vs. ground condition. (H) Rratios of neuron area, neurite area and neurite length show how neurons adapt to simulated microgravity throughout the exposure time. Paired two-tailed Student’s *t*-test and standard deviation bars are shown. 1, *p*<0.05 RPM 1 h compared to GC 1 h; 2, *p*<0.05 RPM 24 h compared to GC 24 h; 3, *p*<0.05 RPM 10 days compared to GC 10 days. GC = ground condition; RPM = Random Positioning Machine.

Neurons cultured for 10 days under ground conditions and thereafter exposed to the RPM for 1 h showed an area reduction of 24% per neuron compared to their respective controls, whereas neurons exposed for 24 h showed a significant area reduction of 14% (Fig. 2A, B and H) per neuron. In neurons exposed for 10 days the 6% area reduction was not significant (Fig. 2A, B and H). Similar effects of microgravity exposure were observed on the neurite area and length per neuron, both of which were significantly reduced after short- to middle-term exposures, but not after long-term exposure (Fig. 2C, D and H). These data therefore indicate that from the initial reduction in neuron/neurite area, which occurred during the first hour of RPM exposure, cells adapted to the new gravity condition over time. Additionally, analyses on neurite network density, estimated as neurite area or neurite length per image, showed a decrease in network density after exposure to the RPM for 1 h or 24 h, whereas networks exposed for 10 days showed partial adaptation (Fig. 2A, E, F and H). Interestingly, in both single neurons and neurite networks the adaptation of the neurite length in simulated microgravity was delayed (Fig. 2H) indicating that the recovery of the neurite area after 24 h should be the result of partial thickening of the neurites (Fig. S4). Furthermore, analysis on neuron density determined in number of neurons per cm^2^ did not show major changes over the different exposure times to simulated microgravity compared to the respective controls (Fig. 2G).

#### Changes in soma morphology and *β*-tubulin isotype 3 distribution in neurons

Microtubules are cytoskeleton elements organized in substructures as protofilaments made by α- and β- tubulin monomers. They are involved in the internal transport, locomotion and cell shape. In particular, microtubules together with microfilaments and intermediate filaments determine the cell architecture which explains how cell shape and other mechanisms are controlled and respond to mechanical forces [41]. Microtubules are under a continuous turn-over of polymerization and depolymerization of their arborization, also known as “treadmilling”, and the dynamic activity is linked to the cell function or to the intracellular or extracellular environment. Staining the cells for the β-tub 3, one of the components of microtubules, allowed us to analyze the distribution of the cytoskeletal protein into neurons exposed to simulated microgravity and therefore estimate variations in the protein distribution between somas and neurites.

**Figure 3:**
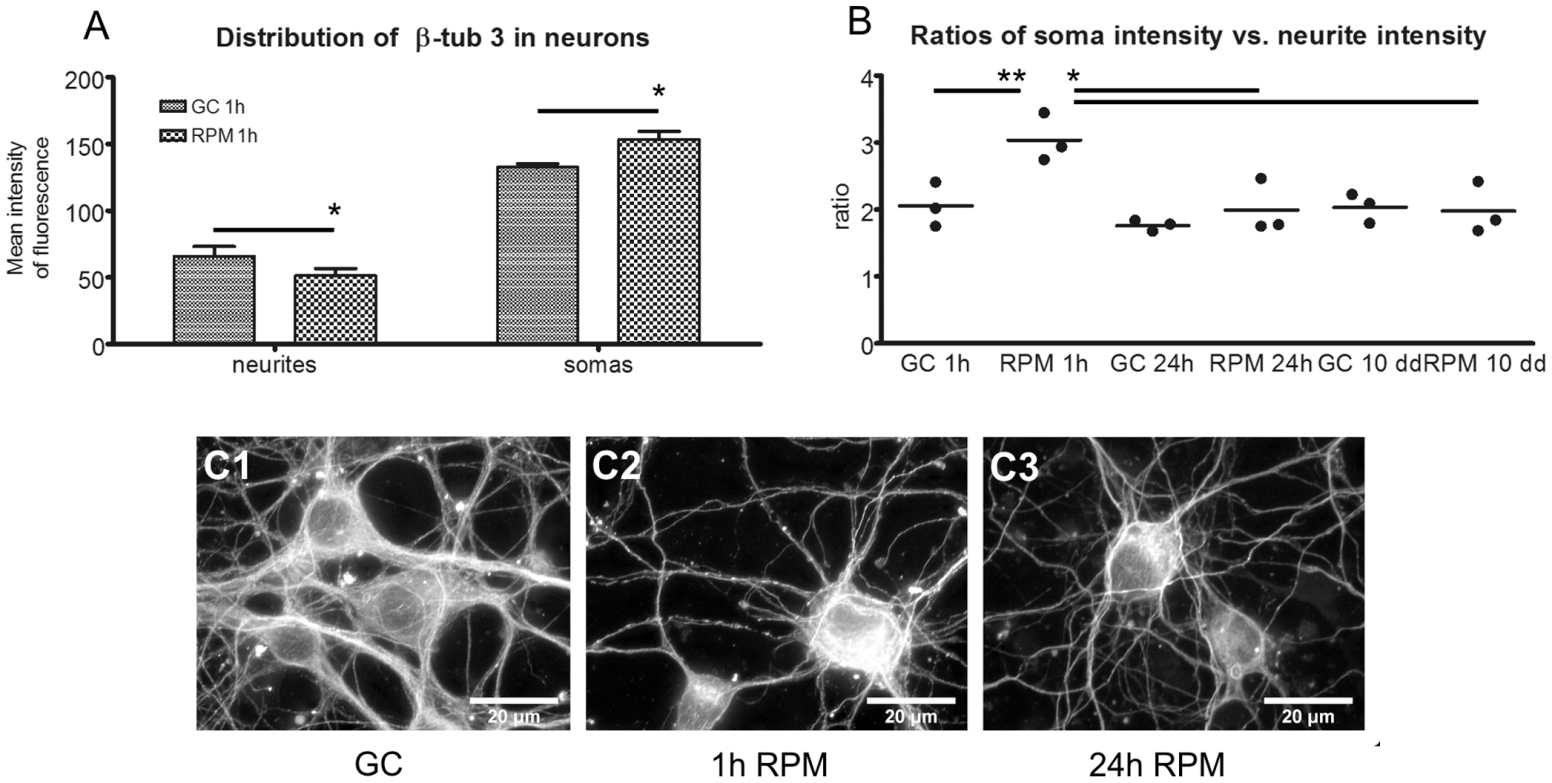
Distribution of β-tubulin isotype 3 (β-tub 3) under simulated microgravity. (A) Distribution of β-tub 3 between somas and neurites in neurons exposed for 1 h to RPM (neurite *p* = 0.029; soma *p* = 0.038). (B) Soma intensity vs. neurite intensity ratios in neurons exposed for 1 hour, 24 hours and 10 days to the RPM compared to the respective ground condition controls. Statistical analysis show a difference between GC 1 h vs. RPM 1 h (*p* = 0.0012) and RPM 1 h vs. rpm 24 h vs. RPM 10 days (*p*<0.05). (C 1-2-3) Higher magnification of neurons show the morphological and fluorescence intensity differences at the soma and neurite levels between exposed and non-exposed samples. One way Anova, Paired two-tailed Student’s *t*-test and standard deviation bars are shown. GC = ground condition; RPM = Random Positioning Machine.

Typically, in neurons exposed to ground conditions, β-tub 3 mean intensity in somas was twice as high as in the neurites (Fig. 3A-B-C1). However, after exposure to the RPM for 1 h, the mean intensity of the fluorescence signal decreased in the neurites (*p* = 0.029) whereas it increased in the soma (*p* = 0.039) (Fig. 3A–C2) which suggests a redistribution of microtubules in the cells upon short-term exposure to microgravity. In contrast, cells exposed for 24 h or 10 days did not show any difference in β-tub 3 distribution compared to their respective controls indicating that the cells had adapted to their new environment (Fig. 3B–C3).

**Figure 4:**
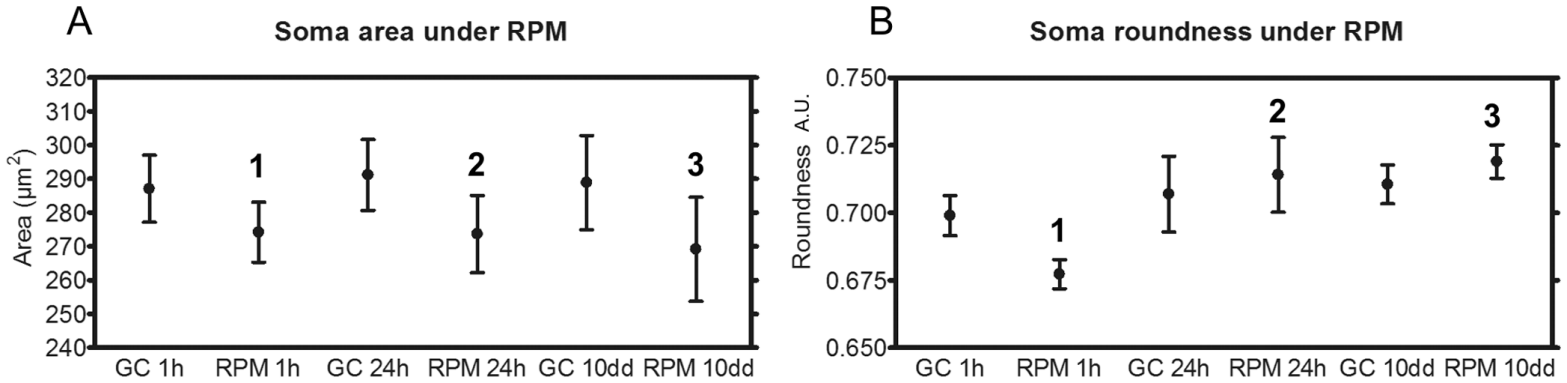
Soma characteristics in neurons exposed for 1 hour, 24 hours and 10 days to the RPM compared to their respective controls. (A) Size of somas in neuron cultures exposed to RPM for 1, 24 hours or 10 days and their respective controls. (B) Roundness of somas in neuron cultures exposed to RPM for 1, 24 hours or 10 days and their respective controls. Paired two-tailed Student’s *t*-test and standard deviation bars are shown. 1, *p*<0.05 RPM 1 h compared to GC 1 h; 2, *p*<0.05 RPM 24 h compared to GC 24 h; 3, *p*<0.05 RPM 10 days compared to GC 10 days. GC = ground condition; RPM = Random Positioning Machine.

Since a redistribution of microtubules may affect the cellular morphology, we also measured the size as well as the shape of the somas. We observed that, at all time points, exposure to microgravity led to a significant reduction of the soma size (Fig. 4A). The shape of each soma was attributed a certain roundness coefficient (ratio between longest and smallest diameter used to determine how far from a perfect circle the shape is), and we found that the roundness was reduced in short-term exposed cells, whereas it was increased in middle- and long-term exposed cells (Fig. 4B).

### Neuronal Network Recovery after Simulated Microgravity

#### Neuron and neurite recovery after simulated microgravity

In order to understand whether microgravity induces permanent or temporary morphological changes, neuronal cultures were exposed again to ground conditions for 24 and 72 hours after having been exposed to simulated microgravity. We analyzed the neuron area, the neurite area and length per image and per neuron as well as the morphological parameters of the somas (size and roundness) to evaluate the recovery after RPM exposure.

**Figure 5:**
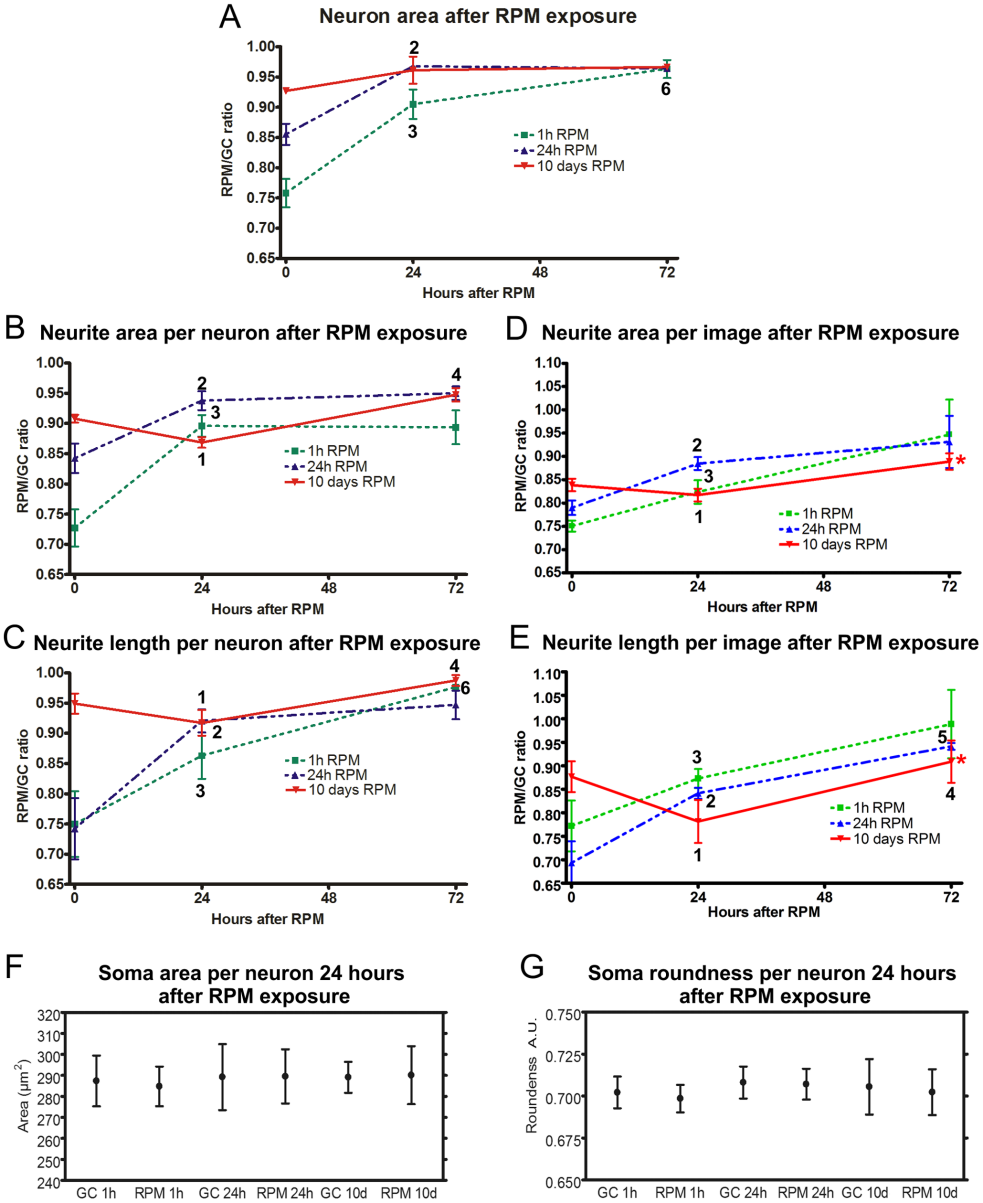
Recovery in re-established ground conditions of neuronal networks and neurons previously exposed to simulated microgravity. (A) Recovery dynamics of area of single neurons after RPM as expressed in ratios of RPM vs. ground control exposed cultures. (B) Recovery dynamics of neurite area per neuron after RPM as expressed in ratios of RPM vs. ground control exposed cultures. (C) Recovery dynamics of neurite length per neuron after RPM as expressed in ratios of RPM vs. ground control exposed cultures. (D) Recovery dynamics of neurite network area per image after RPM as expressed in ratios of RPM vs. ground control exposed cultures. (E) Recovery dynamics of neurite metwork length per image after RPM as expressed in ratios of RPM vs. ground control exposed cultures. (F) Size of somas in neuron cultures previously exposed to RPM and having recovered for 24 hours in ground conditions and their respective controls. (G) Roundness of somas in neuron cultures previously exposed to RPM and having recovered for 24 hours in ground conditions and their respective controls. Paired two-tailed Student’s *t*-test and standard deviation bars are shown.*, *p*<0.05 raw data 72 h RPM vs. raw data 72 h GC; 1, *p*<0.05 24 h vs. 0 h of neuron area after 10 days of RPM; 2, *p*<0.05 24 h vs 0 h of neurite area after 24 h of RPM; 3, *p*<0.05 24 h vs. 0 h of neurite length after 1 h of RPM; 4, *p*<0.05 72 h vs. 24 h of neuron area after 10 days of RPM; 5, *p*<0.05 72 h vs 24 h of neurite area after 24 h of RPM; 6, *p*<0.05 72 h vs. 24 h of neurite length after 1 h of RPM. GC = ground condition; RPM = Random Positioning Machine.

Neurons exposed to simulated microgravity for 1 h showed a significant area increase from 75 to 90% of the respective controls during 24 h of recovery in ground conditions. In the following hours of recovery they reached 96% of their respective controls (Fig. 5A). A similar area increase was also observed in neurons that had been exposed to the RPM for 24 h (Fig. 5A). Neurons exposed for 10 days to RPM did not show any statistical area increase (Fig. 5A). Also the area of neurites of single neurons exposed for 1 h and 24 h to the RPM showed a significant increase in the first 24 h of recovery in ground conditions, whereas neurons exposed to simulated microgravity for 10 days showed a statistical reduction of 4% (from 91% to 87%) in neurite area per neuron within the first 24 h of recovery and reaching 95% of the corresponding controls 72 h after RPM (Fig. 5B). Similar behavior was observed on neurite length per single neurons (Fig. 5C). Furthermore, analyses of neurite network density per image, expressed in neurite area and length, showed a similar recovery such as single neurons (Fig. 5D, E). Additionally, neurite networks having recovered in re-established ground conditions after exposure to simulated microgravity for 10 days showed statistical difference in area and length compared to the respective controls (Fig. 5D, E).

#### Morphology of somas after simulated microgravity

As previously mentioned, somas of neurons exposed to simulated microgravity presented morphological alterations such as a reduction in size and changes in roundness. After being exposed to ground conditions for 24 h following simulated microgravity, these effects were completely reversed for short-, middle- as well as long-term exposed neuronal cultures (Fig. 5F, G), indicating a fast full recovery of the soma morphology.

### Changes in Cell Viability

It is known that apoptosis can be induced by stress when cells are exposed to non-physiological conditions. In order to understand whether neuron viability was altered by exposure to simulated microgravity and throughout the following recovery in ground conditions, neuron cultures were analyzed using the Ann V - PI assay.

**Figure 6:**
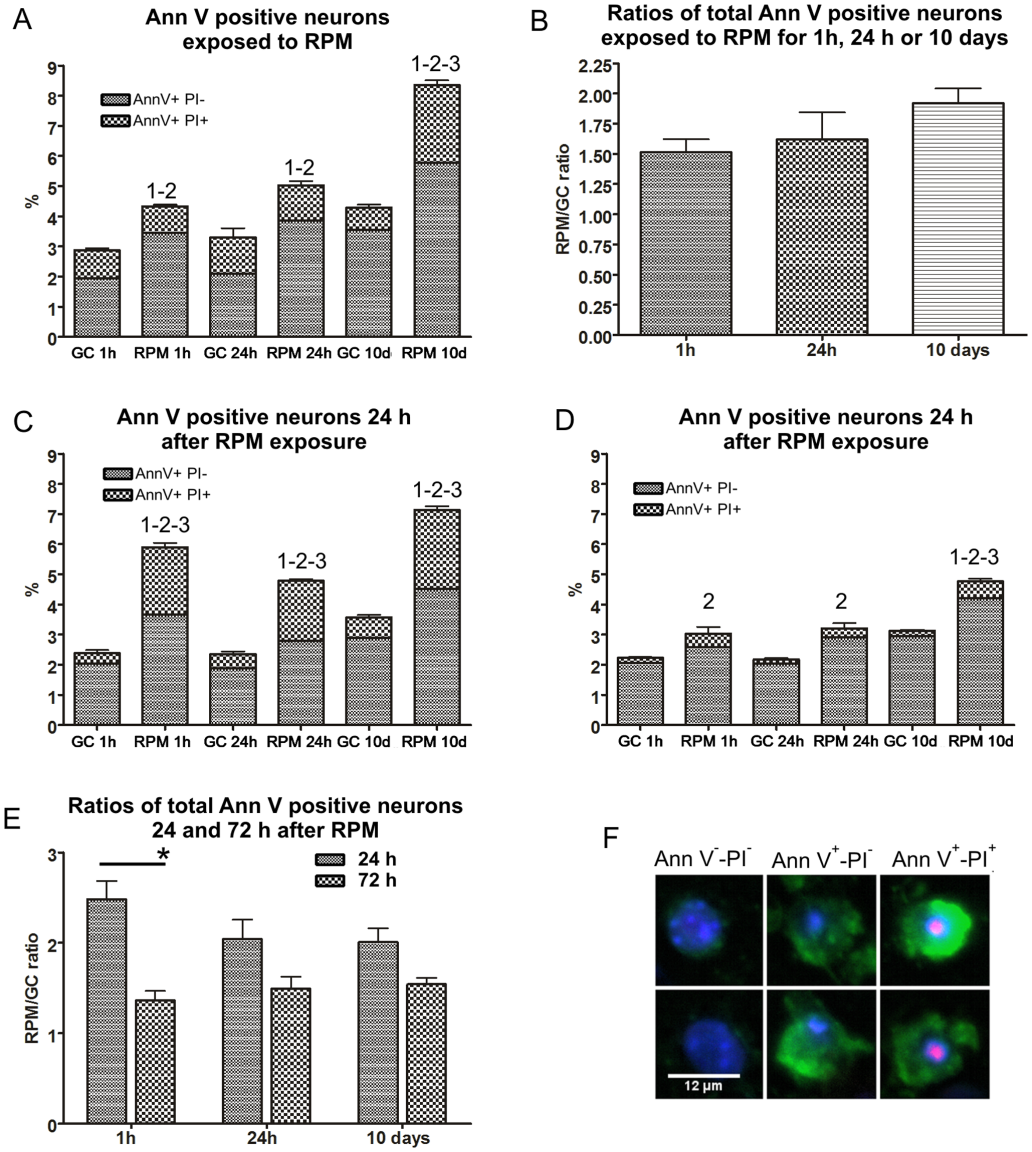
Altered viability induced during and after simulated microgravity exposure. (A) Percentage of Ann V^+^-PI^−^ and Ann V^+^-PI^+^ cells in neuronal network induced after 1 hour, 24 hours or 10 days of simulated microgravity. (B) RPM/GC ratios of total Ann V^+^ percentages of neuron cultures exposed and not exposed to simulated microgravity. (C) Percentages of Ann V^+^-PI^−^ and Ann V^+^-PI^+^ cells in neuron cultures exposed to RPM and having recovered for 24 h in ground conditions. (D) Percentages of Ann V^+^-PI^−^ and Ann V^+^-PI^+^ cells in neuron cultures exposed to RPM and having recovered for 72 h in ground conditions. (E) RPM/GC ratios of total Ann V^+^ percentages of neuron cultures recovered for 24 and 72 hours after simulated microgravity exposure. * = *p*<0.05 24 h compared to 72 h. (F) Neurons stained with Annexin V-FITC/PI/Hoechst (green/red/blue) and observed under fluorescence microscope. Ann V-FITC^−/^PI^−/^Hoechst^+^ are considered healthy cells, V-FITC^+^/PI^−/^Hoechst^+^ are considered early apoptotic cells and Ann V-FITC^+^/PI^+^/Hoechst^+^ are considered late apoptotic or necrotic cells. Paired two-tailed Student’s *t*-test and standard deviation bars are shown. 1, *p*<0.05 Ann V^+^-PI^−^ RPM compared to GC; 2, *p*<0.05 Ann V^+^-PI^+^ RPM compared to GC; 3, *p*<0.05 total Ann V^+^ RPM compared to GC. GC = ground conditions; RPM = Random Positioning Machine.

The percentages of total Ann V positive neurons (total Ann V^+^ = Ann V^+^-PI^−^ +Ann V^+^-PI^+^) in cultures exposed to RPM for 1 h, 24 h and 10 days increased between 1.5 and 2 times compared to the respective controls (Fig. 6A–B). Interestingly, in cultures exposed for 10 days to RPM conditions the number of Ann V^+^-PI^+^ neurons was 3 times more elevated than the controls (Fig. 6A). This increase in Ann V^+^-PI^+^ cells was not observed in cultures exposed for 1 and 24 hours to the RPM (Fig. 6A). Furthermore the rate of total Ann V^+^ neurons (RPM/GC) did not change between short-, middle- and long-term exposure to simulated microgravity (Fig. 6A).

Neuron cultures exposed to the RPM and having recovered for 24 h showed an increase in percentage of total Ann V^+^ cells over 2 times more elevated than the respective controls. Furthermore, an increase in percentage of late apoptotic or necrotic (Ann V^+^-PI^+^) cells was observed in neuron cultures previously exposed for 1 h, 24 h and 10 days to RPM (Fig. 6C). Seventy-two hours after exposure to RPM, only neurons exposed for 10 days still showed statistical differences in both total Ann V^+^and only Ann V^+^-PI^+^ cells compared to the controls (Fig. 6D).

Moreover, it was observed that RPM/GC ratios of total Ann V^+^ showed a statistical decrease between 24 and 72 hours after 1 h RPM exposure (Fig. 6E).

## Discussion

In this study, we investigated morphological changes of well-connected primary neuronal networks cultured under simulated microgravity conditions using the RPM to test whether simulated microgravity can affect neuronal morphology and to establish a possible link with physiological changes that are seen in astronauts during space flight.

### Cellular and Neurite Network Response to the Modified Gravity

In our study we observed that, within the first hour of simulated microgravity exposure, cultured single neurons exhibited a reduction of neurite length, size and roundness of somas indicating shrinkage of the cell. Concomitantly, the area and the length of neurite networks were also affected by the RPM environment. Moreover, β-tub 3 fluorescence intensity analyses showed that 1 h of exposure to microgravity induced changes in the microtubule distribution from the neurites to the soma. This is in accordance with other investigations on the cytoskeleton of cells exposed to short-term microgravity conditions, which described changes of microtubules, microfilaments and intermediate filaments [17], [18], [42]. Additionally, concomitant to microgravity-induced cytoskeletal changes [17], [18], cell area reduction occurred as well [7]. Furthermore, the observed reduction of the roundness of somas, which occurred within the first hour of simulated microgravity seems to be a transitive stage before reaching the final stage in which neuron bodies of cells subjected to simulated microgravity were rounder compared to the controls already at 24 h of RPM.

Studies on cell motility reported a drastic reduction of cell locomotion [7], [17]. On the contrary, in our neuronal cultures, one of the responses observed in modulated gravity is that these cells do not lose their intrinsic property to extend neurites and search for contacts with other neurons. In fact, neurons as well as neuronal networks are initially affected by simulated microgravity and they reacted by increasing area and length of neurites throughout long RPM exposure (Fig. 2B–H). Despite the high neuron adaptation to simulated microgravity during long-term RPM exposure, the partial adaptation of neurite network to simulated microgravity could be explained by the observed increase of apoptosis over long-term exposure.

Within this study, we observed in both single neurons as well as neurite networks that most of the effects of short-term exposure to microgravity are attenuated in middle- and long-term exposed neurons, indicating that the cells adapt to the new gravity conditions. On the other hand, neuron recovery after long-term exposure showed a different re-adaptation to the ground conditions compared to short- and middle-term RPM exposure. After long-term RPM exposure (10 days), we observed in both single neurons and neurite networks an initial decrease in the area and the length of neurites during the first 24 hours of recovery followed by an increase after 72 hours of recovery reaching again almost the normality (Fig. 5B, C).

### Viability of Mature Neurons under Gravitational Changes

As reported in several studies, apoptosis and neuroplasticity (neurogenesis and neuronal network remodeling) are in stable equilibrium. An increase in one may trigger the other and *vice versa* [43]. *In vivo* studies reported that environmental as well as endogenous factors not only decrease neurogenesis but can also induce apoptosis [44], [45]. Indeed, several experiments showed that simulated microgravity induces apoptosis in different cell types [7], [10], [26], [46]. Within our experiments, cell death investigation revealed an increase of total Ann V^+^ cells after exposure to simulated microgravity.

The observed increase of cells positive for the apoptosis markers (total Ann V^+^) within 24 h of recovery after short- and middle-term exposure to microgravity (Fig. 6C) might be due to the fact that, under simulated microgravity, some neurons may have lost their connections as a consequence of the reduction in neurite length. Furthermore, an increase of late apoptotic and necrotic (Ann V^+^-PI^+^) neurons was observed within the first 24 h of recovery after RPM which might induce gaps in the networks. Nevertheless, the number of apoptosis marker positive cells (total Ann V^+^) decreased 72 h after RPM exposure (Fig. 6D, 1–24 h) allowing to re-establish the equilibrium between cell death and neuroplasticity.

Despite the neuron adaptation to simulated microgravity during long-term exposure, we observed an increase in Ann V^+^-PI^+^ neurons in long-term exposed cultures (Fig. 6A), but not in short- or middle-term exposed cultures. These results suggest that the increased percentage of late apoptosis could be the cause of the partial adaptation of neurite networks to simulated microgravity during long-term exposure. Within the first 24 h of recovery after long-term RPM exposure, neuronal networks showed reduction in neurite length and area (Fig. 5A, B), while the level of total Ann V^+^ cells was still higher than the control after 72 h of recovery. Furthermore, the higher percentage of late apoptotic or necrotic cells observed within the 24 h of recovery in ground conditions after RPM (Fig. 6C) might be linked to neuronal network changes observed at the same time point, inducing a delay in the neuronal network recovery observed 72 h after RPM (Fig. 5A, B). Similar effects were reported in studies on skeletal muscles of rat exposed 12.5 days to real space conditions, where fiber necrosis and degeneration of motor innervation were observed a few days after landing [47].

### Neuroplasticity under Space Conditions

It was reported that environmental changes increase the activity of neuroplasticity [48]. In fact, during the first days in space, the nervous system of astronauts is forced to develop new interpretations of the stimuli and to develop alternative adjustment strategies to compensate for the altered incoming stimuli [49]. Behavioral changes, alterations in neuronal activity, structural and biochemical changes were reported during spaceflight [11]. In real space conditions, neuronal network plasticity is modulated by the gravitational changes as well as cosmic radiations, both could influence the integrity and the neuronal network remodeling reducing astronauts capability to perform daily activities [12]. In this study we provided new data on *in vitro* neuronal network changes during short- and middle-term exposure to simulated microgravity and on their partial adaptation over a period of 10 days. This seems to be in agreement with the behavioral tests performed on mice exposed for 91 days to the ISS environment and in which a quick learning in how the mice dealt with the new gravity conditions using the grid to grasp and direct their movements was reported [32].

In this study, we observed an increased level of apoptotic neurons within 10 days of RPM, probably due to the stress induced by simulated microgravity, which might induce a reduction of the neurite network density resulting in an increase of neuroplasticity activity in order to re-establish the lost connections. These results could be the response to an increase of neuroplasticity activity in the neuronal network as reported by the up-regulation of genes related to axonal guidance, branching of neurites and long-term potentiation observed in genome expression analyses on the whole brain tissue of mice recently exposed to ISS environment [32]. Additionally, in line with the re-interpretation of input coming from gravity sensor organs, a high neuroplasticity activity at the level of gravity sensitive hair cells in rats exposed to space conditions during the Space Flight Science was reported [30]. Furthermore, as a consequence of partial muscular atrophy observed in rats subjected to a 3-month hindlimb-upload method to simulate microgravity, an increase in the number of axon terminals was found in some neuromuscular synapses related to active spinal motor-neurons [50].

The observed neuroplasticity (network remodeling and/or apoptosis) might be a factor contributing to changes in brain homeostasis. We provided here a cellular evidence of neuronal network remodeling and neuron adaptation following a stress due to changes in gravity conditions such as the ones experienced by astronauts in space, although neuron cells represent just one piece of the puzzling central nervous system. Nevertheless, we believe that our system is an appropriate model for testing the effects of different space conditions and to better understand the related mechanisms that may compromise the structure and function of the neuronal network.

### Conclusions

With this study we contribute to increase the knowledge in space biology. Overall, our experimental setup was designed in order to shed light on the effects of space conditions such as microgravity on established neuronal networks by analyzing its density as well as neuron morphology, cytoskeleton and viability of neurons after short-, middle- and long-term exposure to simulated microgravity. We also investigated the recovery under ground conditions of neuronal networks previously exposed to simulated microgravity.

Obtained results within this investigation underline two different responses related to simulated microgravity exposure time. First, short-term (1 h) exposure to simulated microgravity induces stress in neurons. Reduction in neurite network density, neuron size, alteration in β-tubulin isotype 3 distributions and increase of apoptotic cells are observed in neurons exposed to the RPM for only 1 hour. During recovery post short-term exposure to simulated microgravity, a fast restoration almost reaching the ground morphological state occurred in the neurite network as well as in neurons. Furthermore, the response observed after short-term exposure to reduced gravity might influence the connectivity stimulating neurons to increase the network by producing new neurites to establish new connections over time.

On the contrary, a second type of response was observed during long-term exposure where single neurons reached a high degree of adaptation to simulated microgravity conditions whereas the neurite network was partially adapted, most probably due to an increase of apoptosis. Additionally, single neuron recovery post long-term exposure to RPM was slower whereas the neuronal network had partially recovered. The neuronal network seems to acquire a different physiological state under microgravity conditions requiring a long re-adaptation period during recovery under ground conditions. This response clearly indicates that the highly adapted neurons to simulated microgravity (10 day exposure to RPM) exhibit different physiological cell state than in normal ground control conditions.

Most of the space motion sickness and the space adaptation syndrome symptoms are related to the nervous system which is forced to develop new strategies to interpret the opposite inputs coming from environmental sensors. This adaptation is probably partly based on neuroplasticity activity. In the light of the obtained results, *in vitro* neuronal networks seem to partially adapt to the reduced gravity conditions during long-term exposure. However, to confirm the physiological changes, complementary studies on metabolic pathways, neuronal connectivity and neuronal network activities should be performed. Investigations on mature neuronal networks exposed to both conditions, microgravity and radiation, are necessary to help deciphering the related health risks for the central nervous system in the context of long and deep space travels.

## Supporting Information

Figure S1 **Experimental layout.** Ten day old neuron cultures used for different times of exposure to RPM or ground conditions (GC) for 1 h, 24 h and 10 days. Cells were then fixed immediately after (0 h) or after 24 and 72 h of recovery in GC.
(TIF)

Figure S2 **Annexin V – PI staining on neuronal networks.** From left to right: nuclei staining, Annexin V staining, propidium iodide (PI) staining and merge. (A) Neuronal network without Ann V positive neurons. (B) Neuronal networks with Ann V positive neurons are highlighted in bright green.
(TIF)

Figure S3 **Image processing analysis of Annexin V – PI staining on neuronal networks.** From images of nuclei (A) regions of interest (ROI) related nuclei (B) were determined and counted. From Ann V images (C) neurite network mean intensity, related to the background, was determined after enlarging all ROI’s of nuclei and inverting the obtained selection (D). Twice the background mean intensity was removed from the Ann V images in order to obtain a clear image of Ann V staining into somas (E). To determine if somas were positive or negative for Ann V staining, the external background was determined as previously described (F) and finally mean intensity related to each soma was estimated. As shown in image F, if the mean intensity related to each soma was higher than twice the background mean intensity, somas were considered as positive. Similar procedure was performed to determine negative or positive neurons to propidium iodide staining. Finally, cells were divided in: 1) AnnV-FITC^−/^PI^−/^Hoechst^+^ named Ann V negative, which characterizes normal neurons. 2) AnnV-FITC^+^/PI^−/^Hoechst^+^ named Ann V positive, which characterizes neurons in early apoptosis. 3) AnnV-FITC^+^/PI^+^/Hoechst^+^ named Ann V-PI positive, which characterizes neurons in late apoptosis or necrosis. 4) AnnV-FITC^−/^PI^+^/Hoechst^+^ named PI positive, which characterizes neurons in necrosis or non-neuron cells or decreases.
(TIF)

Figure S4 **Neurite thickness.** Average of neurite thickness determined dividing neurite area by neurite length. No statistical difference was observed with Paired two-tailed Student’s *t*-test and bars represent standard deviation.
(TIF)
