# Supplementary figures and images for: Morphological and Physiological Changes in Mature In Vitro Neuronal Networks towards Exposure to Short-, Middle- or Long-Term Simulated Microgravity
Source: PLoS One. 2013 Sep 16;8(9):e73857. doi: 10.1371/journal.pone.0073857 (PMC3774774; doi:10.1371/journal.pone.0073857)

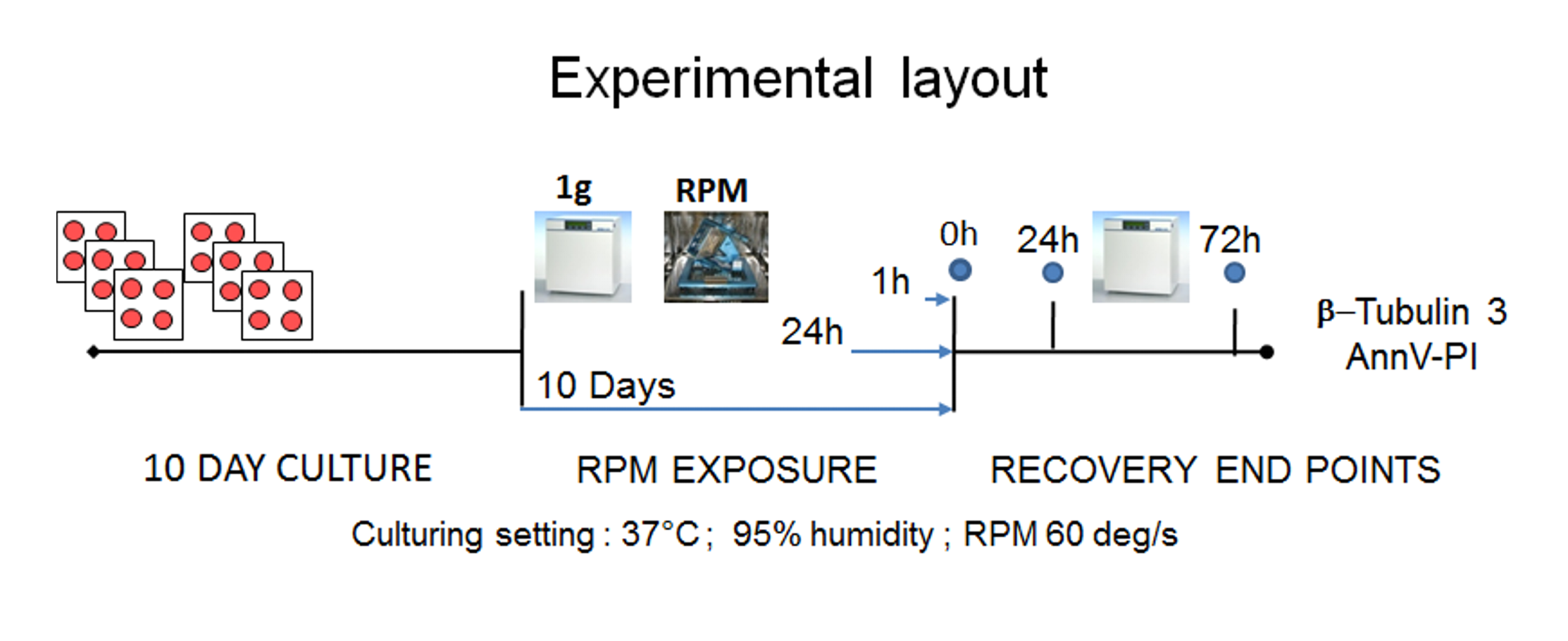

Supplement: Figure S1 — Experimental layout. Ten day old neuron cultures used for different times of exposure to RPM or ground conditions (GC) for 1 h, 24 h and 10 days. Cells were then fixed immediately after (0 h) or after 24 and 72 h of recovery in GC. (TIF) [file pone.0073857.s001.tif]

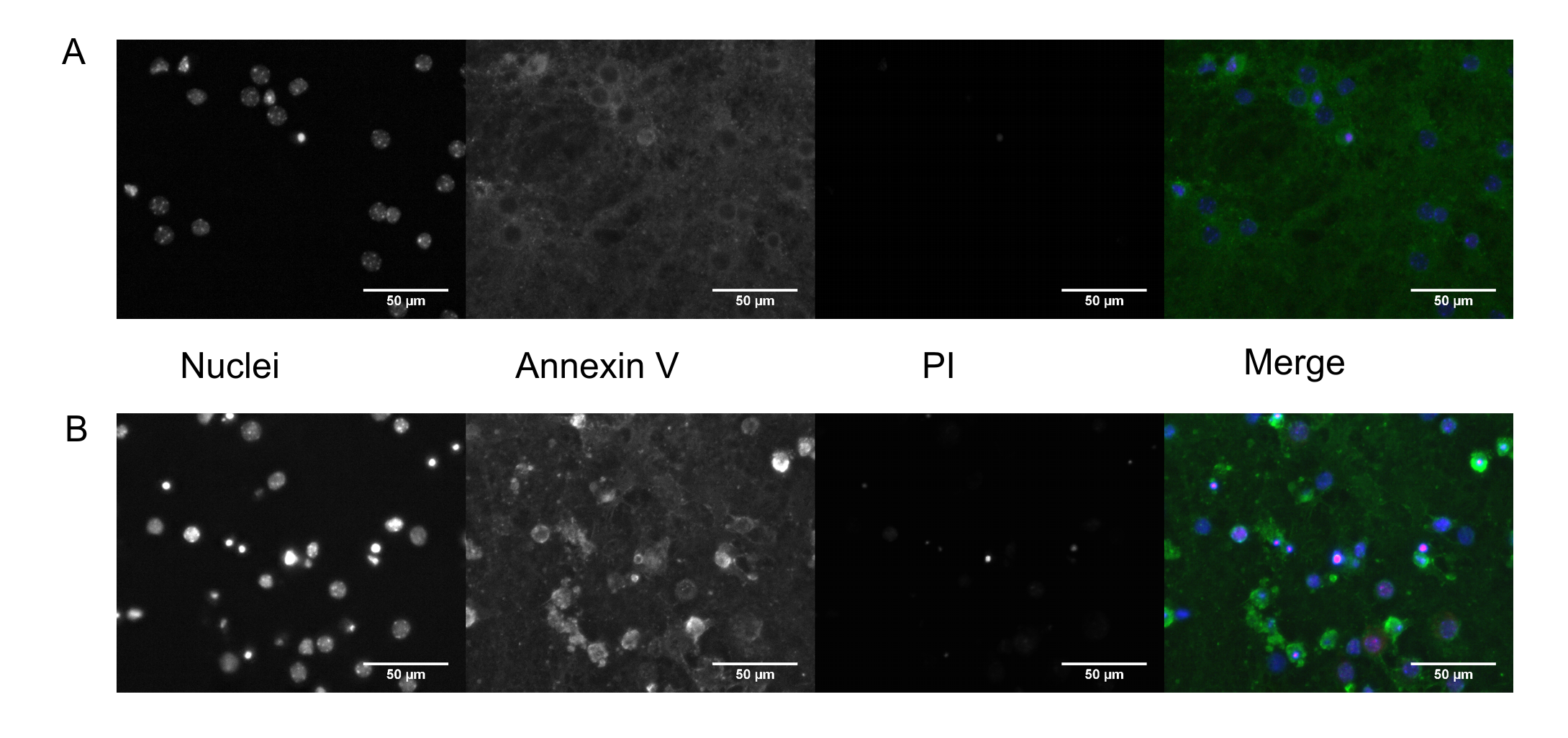

Supplement: Figure S2 — Annexin V – PI staining on neuronal networks. From left to right: nuclei staining, Annexin V staining, propidium iodide (PI) staining and merge. (A) Neuronal network without Ann V positive neurons. (B) Neuronal networks with Ann V positive neurons are highlighted in bright green. (TIF) [file pone.0073857.s002.tif]

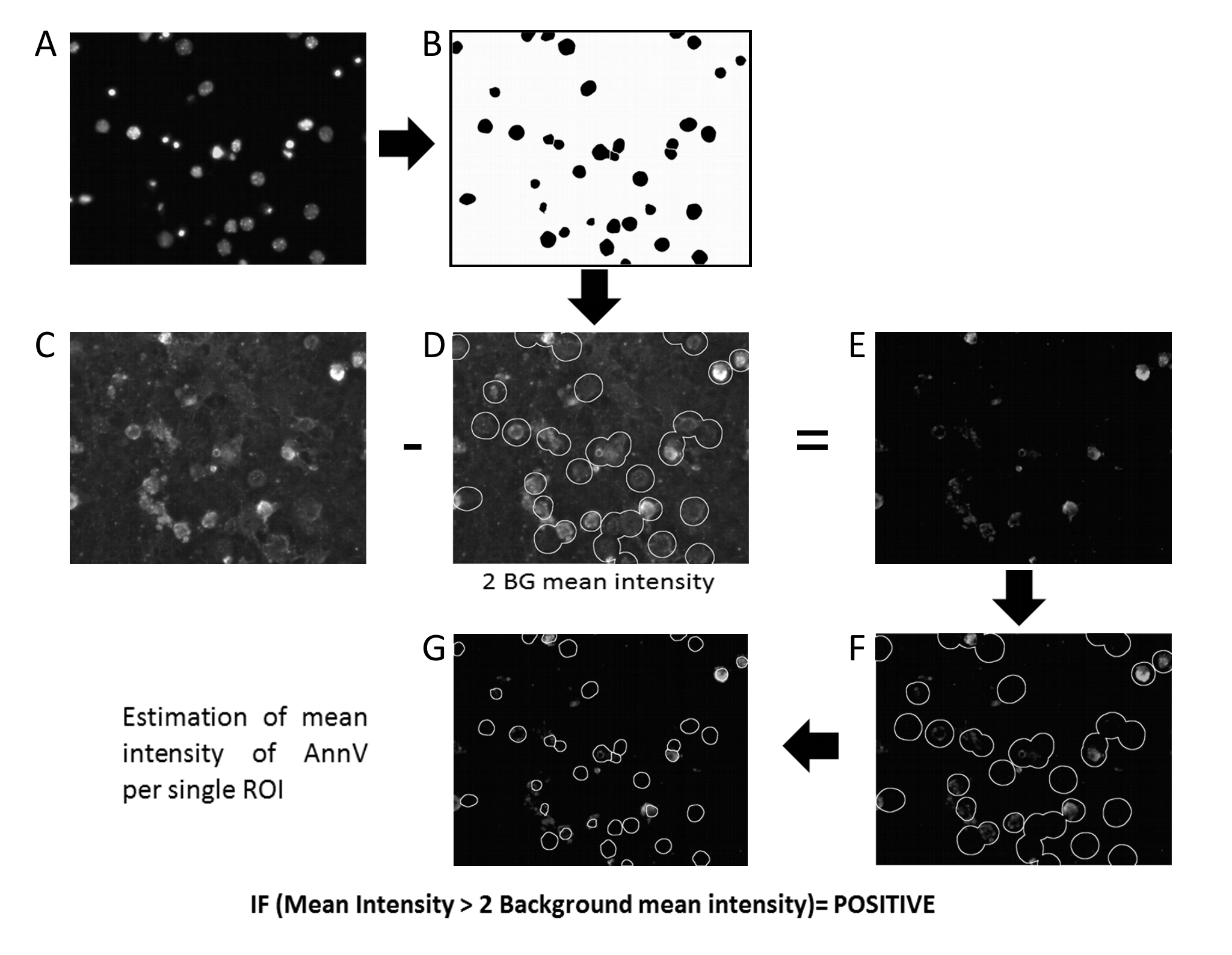

Supplement: Figure S3 — Image processing analysis of Annexin V – PI staining on neuronal networks. From images of nuclei (A) regions of interest (ROI) related nuclei (B) were determined and counted. From Ann V images (C) neurite network mean intensity, related to the background, was determined after enlarging all ROI’s of nuclei and inverting the obtained selection (D). Twice the background mean intensity was removed from the Ann V images in order to obtain a clear image of Ann V staining into somas (E). To determine if somas were positive or negative for Ann V staining, the external background was determined as previously described (F) and finally mean intensity related to each soma was estimated. As shown in image F, if the mean intensity related to each soma was higher than twice the background mean intensity, somas were considered as positive. Similar procedure was performed to determine negative or positive neurons to propidium iodide staining. Finally, cells were divided in: 1) AnnV-FITC−/PI−/Hoechst+ named Ann V negative, which characterizes normal neurons. 2) AnnV-FITC+/PI−/Hoechst+ named Ann V positive, which characterizes neurons in early apoptosis. 3) AnnV-FITC+/PI+/Hoechst+ named Ann V-PI positive, which characterizes neurons in late apoptosis or necrosis. 4) AnnV-FITC−/PI+/Hoechst+ named PI positive, which characterizes neurons in necrosis or non-neuron cells or decreases. (TIF) [file pone.0073857.s003.tif]

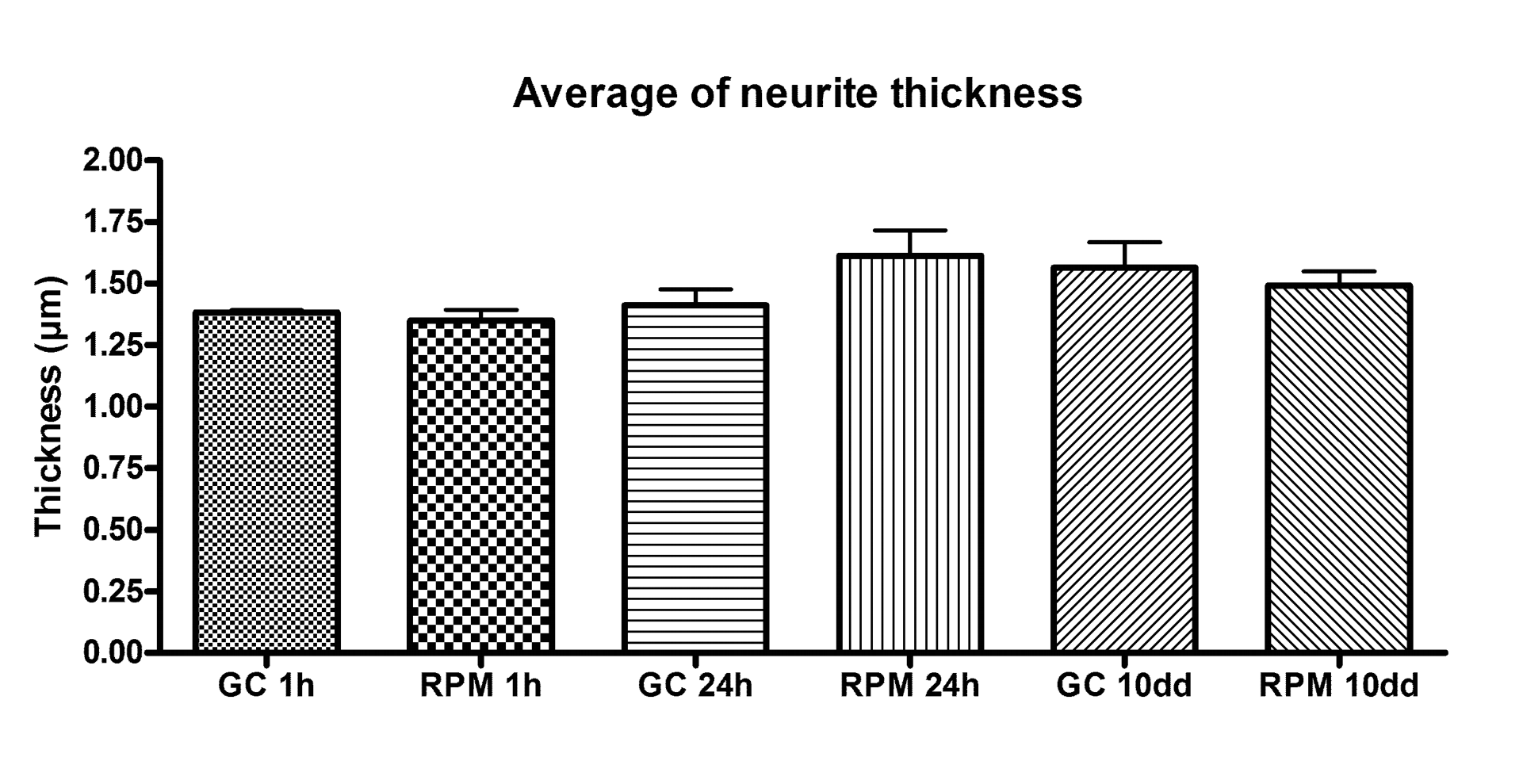

Supplement: Figure S4 — Neurite thickness. Average of neurite thickness determined dividing neurite area by neurite length. No statistical difference was observed with Paired two-tailed Student’s t-test and bars represent standard deviation. (TIF) [file pone.0073857.s004.tif]
